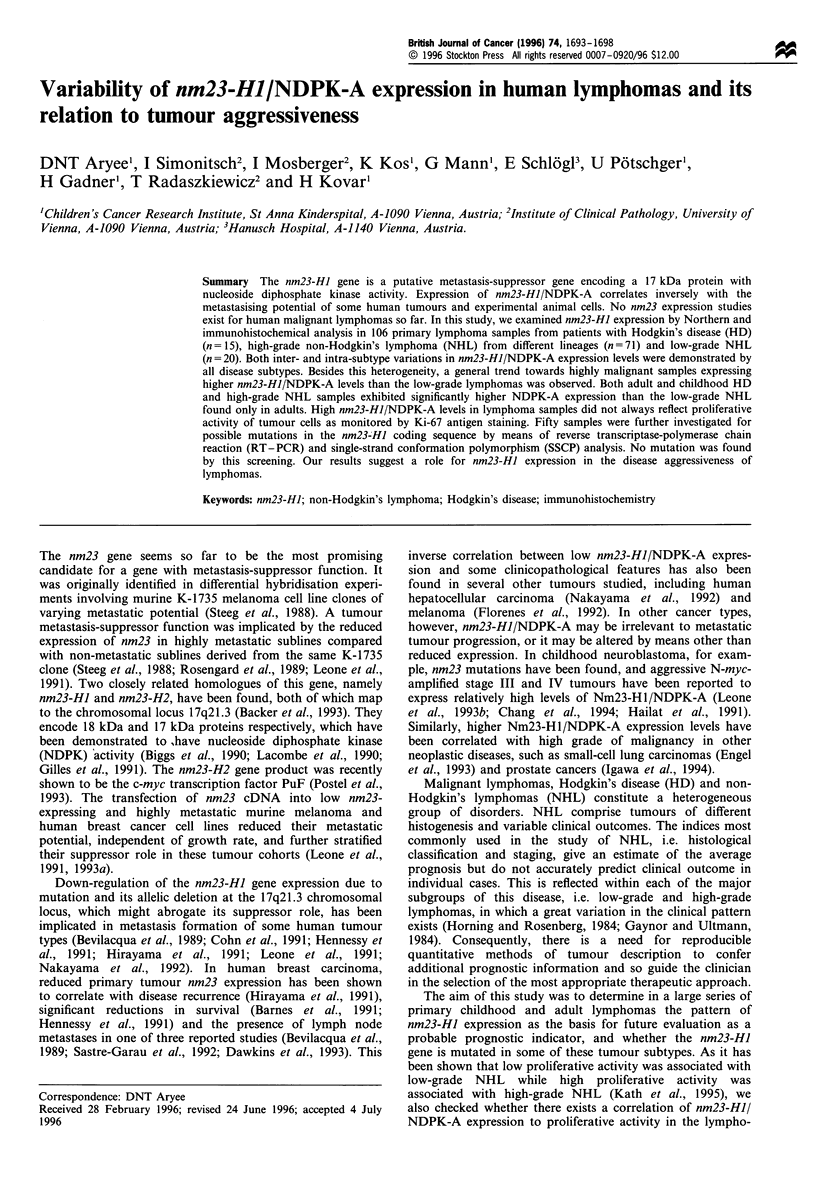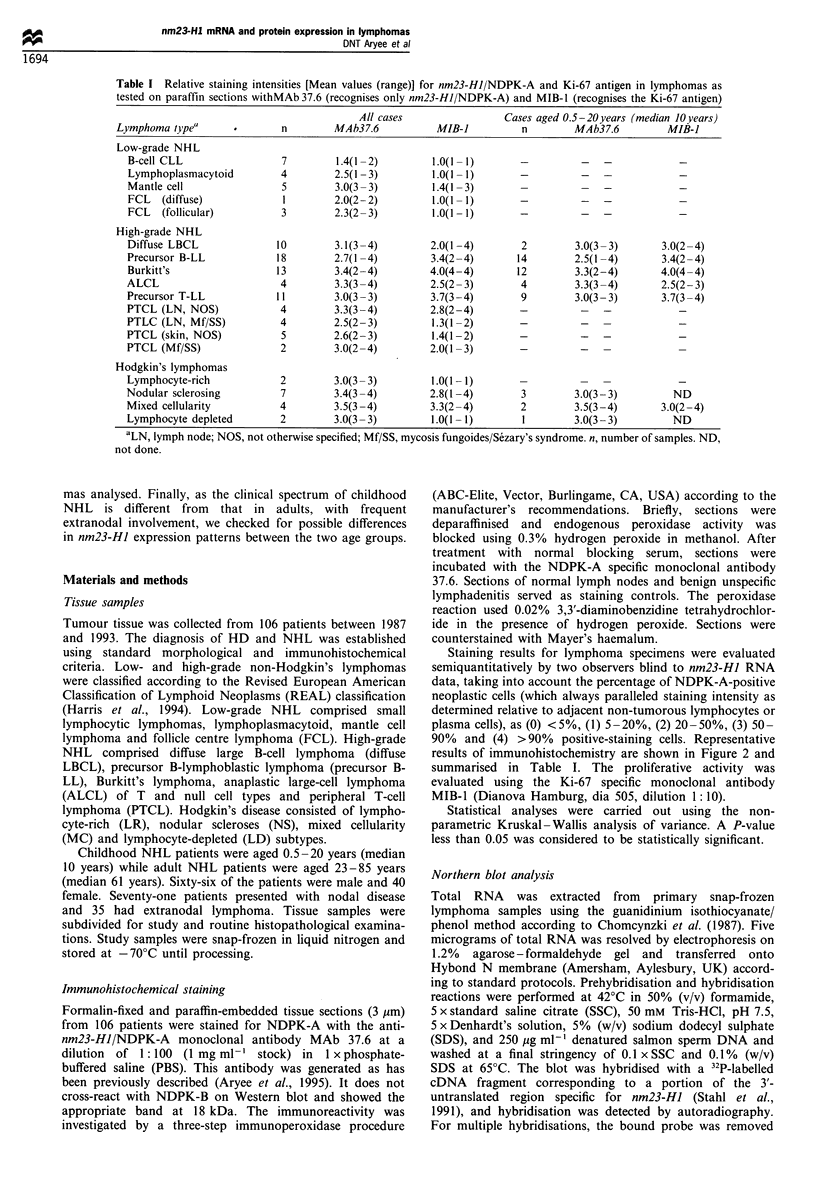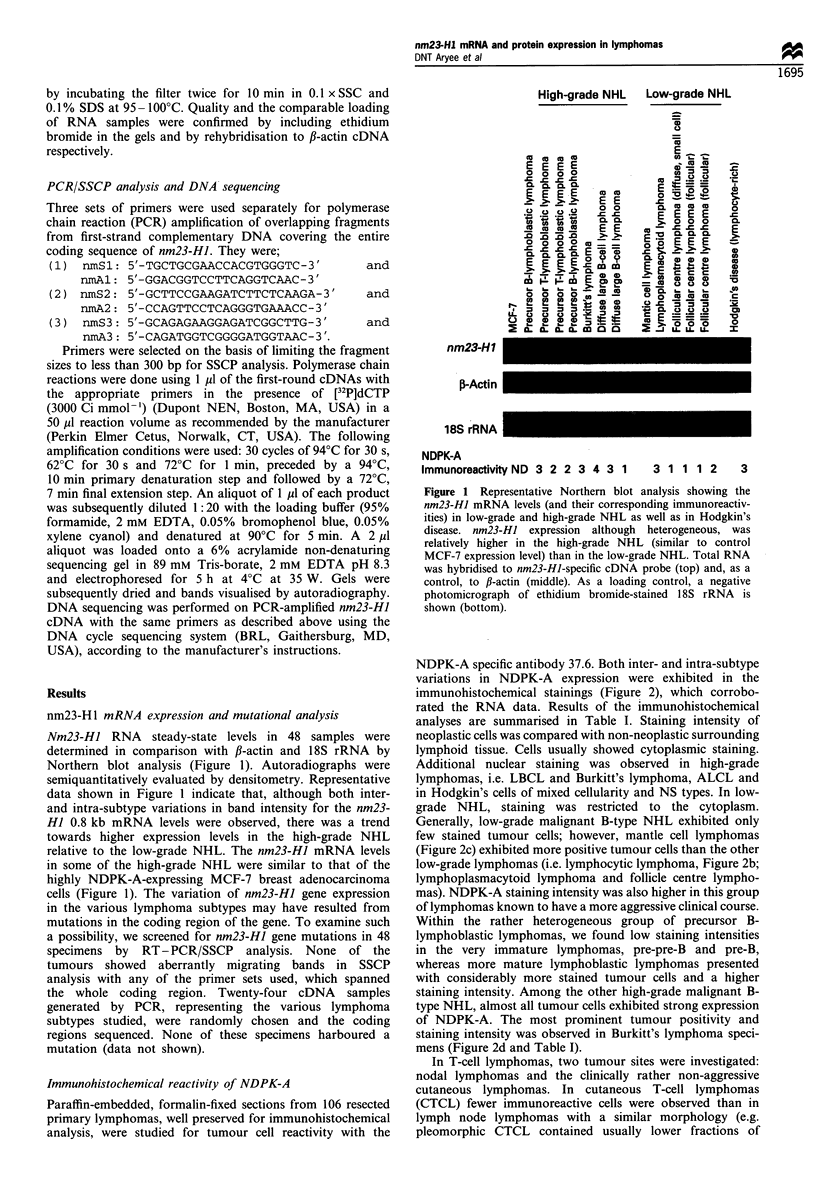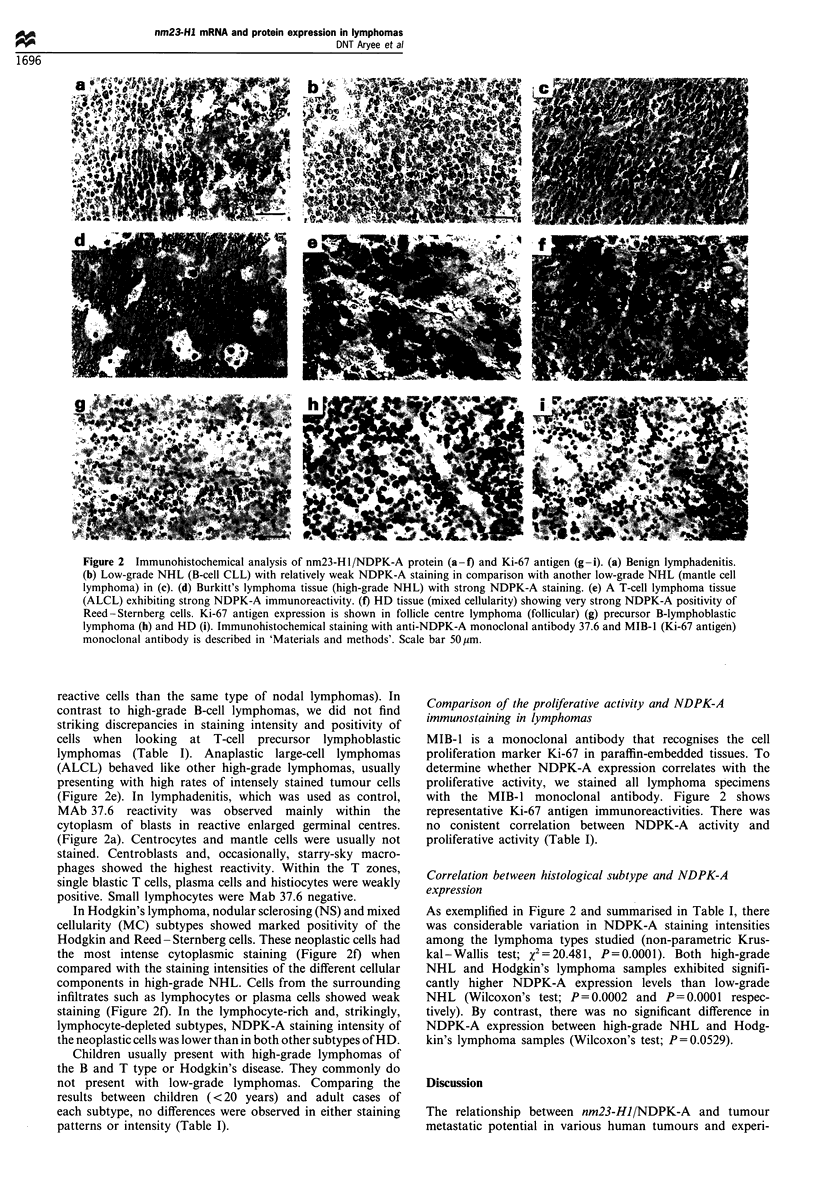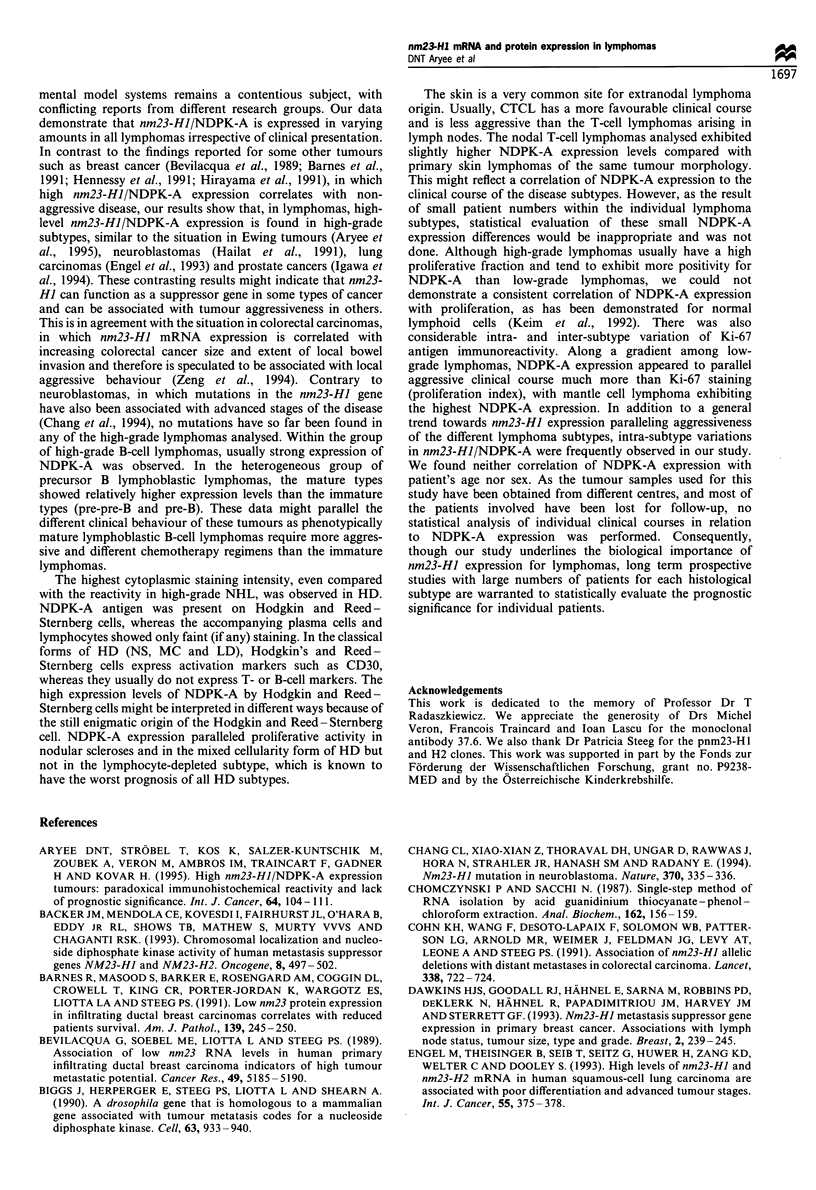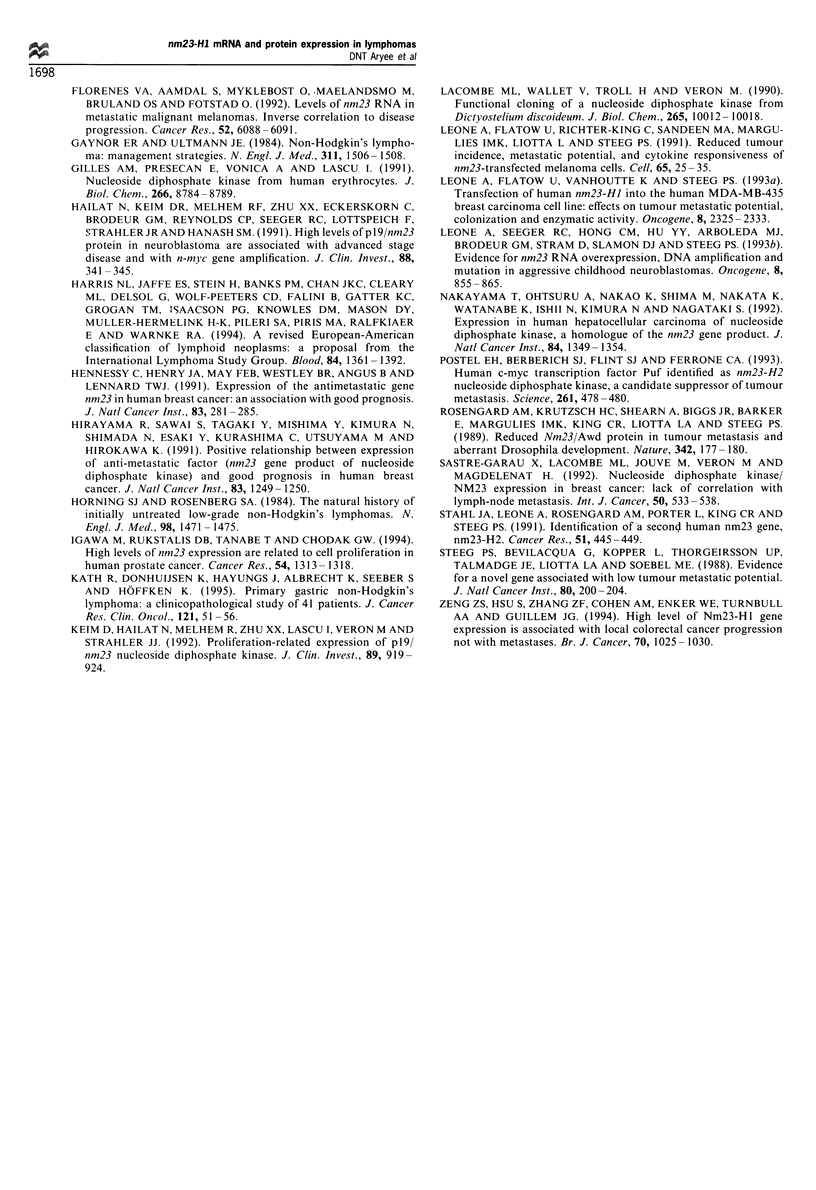# Variability of nm23-H1/NDPK-A expression in human lymphomas and its relation to tumour aggressiveness.

**DOI:** 10.1038/bjc.1996.616

**Published:** 1996-12

**Authors:** D. N. Aryee, I. Simonitsch, I. Mosberger, K. Kos, G. Mann, E. Schlögl, U. Pötschger, H. Gadner, T. Radaszkiewicz, H. Kovar

**Affiliations:** Children's Cancer Research Institute, St Anna Kinderspital, Vienna, Austria.

## Abstract

**Images:**


British Journal of Cancer (1996) 74, 1693-1698
© 1996 Stockton Press All rights reserved 0007-0920/96 $12.00

### Variability of *nm23-H1*/NDPK-A expression in human lymphomas and its relation to tumour aggressiveness

DNT Aryee[1], I Simonitsch[2], I Mosberger[2], K Kos[1], G Mann[1], E Schlögl[3], U Pötschger[1], H Gadner[1], T Radaszkiewicz[2] and H Kovar[1]

[1]*Children's Cancer Research Institute, St Anna Kinderspital, A-1090 Vienna, Austria;* [2]*Institute of Clinical Pathology, University of Vienna, A-1090 Vienna, Austria;* [3]*Hanusch Hospital, A-1140 Vienna, Austria.*

**Summary** The *nm23-H1* gene is a putative metastasis-suppressor gene encoding a 17 kDa protein with nucleoside diphosphate kinase activity. Expression of *nm23-H1*/NDPK-A correlates inversely with the metastasising potential of some human tumours and experimental animal cells. No *nm23* expression studies exist for human malignant lymphomas so far. In this study, we examined *nm23-H1* expression by Northern and immunohistochemical analysis in 106 primary lymphoma samples from patients with Hodgkin's disease (HD) ($n=15$), high-grade non-Hodgkin's lymphoma (NHL) from different lineages ($n=71$) and low-grade NHL ($n=20$). Both inter- and intra-subtype variations in *nm23-H1*/NDPK-A expression levels were demonstrated by all disease subtypes. Besides this heterogeneity, a general trend towards highly malignant samples expressing higher *nm23-H1*/NDPK-A levels than the low-grade lymphomas was observed. Both adult and childhood HD and high-grade NHL samples exhibited significantly higher NDPK-A expression than the low-grade NHL found only in adults. High *nm23-H1*/NDPK-A levels in lymphoma samples did not always reflect proliferative activity of tumour cells as monitored by Ki-67 antigen staining. Fifty samples were further investigated for possible mutations in the *nm23-H1* coding sequence by means of reverse transcriptase-polymerase chain reaction (RT-PCR) and single-strand conformation polymorphism (SSCP) analysis. No mutation was found by this screening. Our results suggest a role for *nm23-H1* expression in the disease aggressiveness of lymphomas.

**Keywords:** *nm23-H1*; non-Hodgkin's lymphoma; Hodgkin's disease; immunohistochemistry

The *nm23* gene seems so far to be the most promising candidate for a gene with metastasis-suppressor function. It was originally identified in differential hybridisation experiments involving murine K-1735 melanoma cell line clones of varying metastatic potential (Steeg *et al.*, 1988). A tumour metastasis-suppressor function was implicated by the reduced expression of *nm23* in highly metastatic sublines compared with non-metastatic sublines derived from the same K-1735 clone (Steeg *et al.*, 1988; Rosengard *et al.*, 1989; Leone *et al.*, 1991). Two closely related homologues of this gene, namely *nm23-H1* and *nm23-H2*, have been found, both of which map to the chromosomal locus 17q21.3 (Backer *et al.*, 1993). They encode 18 kDa and 17 kDa proteins respectively, which have been demonstrated to have nucleoside diphosphate kinase (NDPK) activity (Biggs *et al.*, 1990; Lacombe *et al.*, 1990; Gilles *et al.*, 1991). The *nm23-H2* gene product was recently shown to be the c-*myc* transcription factor PuF (Postel *et al.*, 1993). The transfection of *nm23* cDNA into low *nm23*-expressing and highly metastatic murine melanoma and human breast cancer cell lines reduced their metastatic potential, independent of growth rate, and further stratified their suppressor role in these tumour cohorts (Leone *et al.*, 1991, 1993*a*).

Down-regulation of the *nm23-H1* gene expression due to mutation and its allelic deletion at the 17q21.3 chromosomal locus, which might abrogate its suppressor role, has been implicated in metastasis formation of some human tumour types (Bevilacqua *et al.*, 1989; Cohn *et al.*, 1991; Hennessy *et al.*, 1991; Hirayama *et al.*, 1991; Leone *et al.*, 1991; Nakayama *et al.*, 1992). In human breast carcinoma, reduced primary tumour *nm23* expression has been shown to correlate with disease recurrence (Hirayama *et al.*, 1991), significant reductions in survival (Barnes *et al.*, 1991; Hennessy *et al.*, 1991) and the presence of lymph node metastases in one of three reported studies (Bevilacqua *et al.*, 1989; Sastre-Garau *et al.*, 1992; Dawkins *et al.*, 1993). This inverse correlation between low *nm23-H1*/NDPK-A expression and some clinicopathological features has also been found in several other tumours studied, including human hepatocellular carcinoma (Nakayama *et al.*, 1992) and melanoma (Florenes *et al.*, 1992). In other cancer types, however, *nm23-H1*/NDPK-A may be irrelevant to metastatic tumour progression, or it may be altered by means other than reduced expression. In childhood neuroblastoma, for example, *nm23* mutations have been found, and aggressive N-*myc*-amplified stage III and IV tumours have been reported to express relatively high levels of Nm23-H1/NDPK-A (Leone *et al.*, 1993*b*; Chang *et al.*, 1994; Hailat *et al.*, 1991). Similarly, higher Nm23-H1/NDPK-A expression levels have been correlated with high grade of malignancy in other neoplastic diseases, such as small-cell lung carcinomas (Engel *et al.*, 1993) and prostate cancers (Igawa *et al.*, 1994).

Malignant lymphomas, Hodgkin's disease (HD) and non-Hodgkin's lymphomas (NHL) constitute a heterogeneous group of disorders. NHL comprise tumours of different histogenesis and variable clinical outcomes. The indices most commonly used in the study of NHL, i.e. histological classification and staging, give an estimate of the average prognosis but do not accurately predict clinical outcome in individual cases. This is reflected within each of the major subgroups of this disease, i.e. low-grade and high-grade lymphomas, in which a great variation in the clinical pattern exists (Horning and Rosenberg, 1984; Gaynor and Ultmann, 1984). Consequently, there is a need for reproducible quantitative methods of tumour description to confer additional prognostic information and so guide the clinician in the selection of the most appropriate therapeutic approach.

The aim of this study was to determine in a large series of primary childhood and adult lymphomas the pattern of *nm23-H1* expression as the basis for future evaluation as a probable prognostic indicator, and whether the *nm23-H1* gene is mutated in some of these tumour subtypes. As it has been shown that low proliferative activity was associated with low-grade NHL while high proliferative activity was associated with high-grade NHL (Kath *et al.*, 1995), we also checked whether there exists a correlation of *nm23-H1*/NDPK-A expression to proliferative activity in the lympho-

Correspondence: DNT Aryee
Received 28 February 1996; revised 24 June 1996; accepted 4 July 1996

**Table I** Relative staining intensities [Mean values (range)] for *nm23-H1*/NDPK-A and Ki-67 antigen in lymphomas as tested on paraffin sections withMAb 37.6 (recognises only *nm23-H1*/NDPK-A) and MIB-1 (recognises the Ki-67 antigen)

| Lymphoma type[a] | n | All cases MAb37.6 | MIB-1 | Cases aged 0.5–20 years (median 10 years) n | MAb37.6 | MIB-1 |
|---|---|---|---|---|---|---|
| Low-grade NHL | | | | | | |
| B-cell CLL | 7 | 1.4(1–2) | 1.0(1–1) | – | – – | – |
| Lymphoplasmacytoid | 4 | 2.5(1–3) | 1.0(1–1) | – | – – | – |
| Mantle cell | 5 | 3.0(3–3) | 1.4(1–3) | – | – – | – |
| FCL (diffuse) | 1 | 2.0(2–2) | 1.0(1–1) | – | – – | – |
| FCL (follicular) | 3 | 2.3(2–3) | 1.0(1–1) | – | – – | – |
| High-grade NHL | | | | | | |
| Diffuse LBCL | 10 | 3.1(3–4) | 2.0(1–4) | 2 | 3.0(3–3) | 3.0(2–4) |
| Precursor B-LL | 18 | 2.7(1–4) | 3.4(2–4) | 14 | 2.5(1–4) | 3.4(2–4) |
| Burkitt's | 13 | 3.4(2–4) | 4.0(4–4) | 12 | 3.3(2–4) | 4.0(4–4) |
| ALCL | 4 | 3.3(3–4) | 2.5(2–3) | 4 | 3.3(3–4) | 2.5(2–3) |
| Precursor T-LL | 11 | 3.0(3–3) | 3.7(3–4) | 9 | 3.0(3–3) | 3.7(3–4) |
| PTCL (LN, NOS) | 4 | 3.3(3–4) | 2.8(2–4) | – | – – | – |
| PTLC (LN, Mf/SS) | 4 | 2.5(2–3) | 1.3(1–2) | – | – – | – |
| PTCL (skin, NOS) | 5 | 2.6(2–3) | 1.4(1–2) | – | – – | – |
| PTCL (Mf/SS) | 2 | 3.0(2–4) | 2.0(1–3) | – | – – | – |
| Hodgkin's lymphomas | | | | | | |
| Lymphocyte-rich | 2 | 3.0(3–3) | 1.0(1–1) | – | – – | – |
| Nodular sclerosing | 7 | 3.4(3–4) | 2.8(1–4) | 3 | 3.0(3–3) | ND |
| Mixed cellularity | 4 | 3.5(3–4) | 3.3(2–4) | 2 | 3.5(3–4) | 3.0(2–4) |
| Lymphocyte depleted | 2 | 3.0(3–3) | 1.0(1–1) | 1 | 3.0(3–3) | ND |

[a]LN, lymph node; NOS, not otherwise specified; Mf/SS, mycosis fungoides/Sézary's syndrome. *n*, number of samples. ND, not done.

mas analysed. Finally, as the clinical spectrum of childhood NHL is different from that in adults, with frequent extranodal involvement, we checked for possible differences in *nm23-H1* expression patterns between the two age groups.

### Materials and methods

### *Tissue samples*

Tumour tissue was collected from 106 patients between 1987 and 1993. The diagnosis of HD and NHL was established using standard morphological and immunohistochemical criteria. Low- and high-grade non-Hodgkin's lymphomas were classified according to the Revised European American Classification of Lymphoid Neoplasms (REAL) classification (Harris *et al.*, 1994). Low-grade NHL comprised small lymphocytic lymphomas, lymphoplasmacytoid, mantle cell lymphoma and follicle centre lymphoma (FCL). High-grade NHL comprised diffuse large B-cell lymphoma (diffuse LBCL), precursor B-lymphoblastic lymphoma (precursor B-LL), Burkitt's lymphoma, anaplastic large-cell lymphoma (ALCL) of T and null cell types and peripheral T-cell lymphoma (PTCL). Hodgkin's disease consisted of lymphocyte-rich (LR), nodular scleroses (NS), mixed cellularity (MC) and lymphocyte-depleted (LD) subtypes.

Childhood NHL patients were aged 0.5–20 years (median 10 years) while adult NHL patients were aged 23–85 years (median 61 years). Sixty-six of the patients were male and 40 female. Seventy-one patients presented with nodal disease and 35 had extranodal lymphoma. Tissue samples were subdivided for study and routine histopathological examinations. Study samples were snap-frozen in liquid nitrogen and stored at $-70$°C until processing.

### *Immunohistochemical staining*

Formalin-fixed and paraffin-embedded tissue sections (3 $\mu$m) from 106 patients were stained for NDPK-A with the anti-*nm23-H1*/NDPK-A monoclonal antibody MAb 37.6 at a dilution of 1 : 100 (1 mg ml$^{-1}$ stock) in 1 × phosphate-buffered saline (PBS). This antibody was generated as has been previously described (Aryee *et al.*, 1995). It does not cross-react with NDPK-B on Western blot and showed the appropriate band at 18 kDa. The immunoreactivity was investigated by a three-step immunoperoxidase procedure (ABC-Elite, Vector, Burlingame, CA, USA) according to the manufacturer's recommendations. Briefly, sections were deparaffinised and endogenous peroxidase activity was blocked using 0.3% hydrogen peroxide in methanol. After treatment with normal blocking serum, sections were incubated with the NDPK-A specific monoclonal antibody 37.6. Sections of normal lymph nodes and benign unspecific lymphadenitis served as staining controls. The peroxidase reaction used 0.02% 3,3′-diaminobenzidine tetrahydrochloride in the presence of hydrogen peroxide. Sections were counterstained with Mayer's haemalum.

Staining results for lymphoma specimens were evaluated semiquantitatively by two observers blind to *nm23-H1* RNA data, taking into account the percentage of NDPK-A-positive neoplastic cells (which always paralleled staining intensity as determined relative to adjacent non-tumorous lymphocytes or plasma cells), as (0) <5%, (1) 5–20%, (2) 20–50%, (3) 50–90% and (4) >90% positive-staining cells. Representative results of immunohistochemistry are shown in Figure 2 and summarised in Table I. The proliferative activity was evaluated using the Ki-67 specific monoclonal antibody MIB-1 (Dianova Hamburg, dia 505, dilution 1 : 10).

Statistical analyses were carried out using the non-parametric Kruskal–Wallis analysis of variance. A *P*-value less than 0.05 was considered to be statistically significant.

### *Northern blot analysis*

Total RNA was extracted from primary snap-frozen lymphoma samples using the guanidinium isothiocyanate/phenol method according to Chomcynzki *et al.* (1987). Five micrograms of total RNA was resolved by electrophoresis on 1.2% agarose–formaldehyde gel and transferred onto Hybond N membrane (Amersham, Aylesbury, UK) according to standard protocols. Prehybridisation and hybridisation reactions were performed at 42°C in 50% (v/v) formamide, 5 × standard saline citrate (SSC), 50 mM Tris-HCl, pH 7.5, 5 × Denhardt's solution, 5% (w/v) sodium dodecyl sulphate (SDS), and 250 $\mu$g ml$^{-1}$ denatured salmon sperm DNA and washed at a final stringency of 0.1 × SSC and 0.1% (w/v) SDS at 65°C. The blot was hybridised with a $^{32}$P-labelled cDNA fragment corresponding to a portion of the 3′-untranslated region specific for *nm23-H1* (Stahl *et al.*, 1991), and hybridisation was detected by autoradiography. For multiple hybridisations, the bound probe was removed by incubating the filter twice for 10 min in 0.1 × SSC and 0.1% SDS at 95–100°C. Quality and the comparable loading of RNA samples were confirmed by including ethidium bromide in the gels and by rehybridisation to β-actin cDNA respectively.

*PCR/SSCP analysis and DNA sequencing*

Three sets of primers were used separately for polymerase chain reaction (PCR) amplification of overlapping fragments from first-strand complementary DNA covering the entire coding sequence of *nm23-H1*. They were;

(1) nmS1: 5'-TGCTGCGAACCACGTGGGTC-3' and
nmA1: 5'-GGACGGTCCTTCAGGTCAAC-3'
(2) nmS2: 5'-GCTTCCGAAGATCTTCTCAAGA-3' and
nmA2: 5'-CCAGTTCCTCAGGGTGAAACC-3'
(3) nmS3: 5'-GCAGAGAAGGAGATCGGCTTG-3' and
nmA3: 5'-CAGATGGTCGGGGATGGTAAC-3'.

Primers were selected on the basis of limiting the fragment sizes to less than 300 bp for SSCP analysis. Polymerase chain reactions were done using 1 μl of the first-round cDNAs with the appropriate primers in the presence of [$^{32}$P]dCTP (3000 Ci mmol$^{-1}$) (Dupont NEN, Boston, MA, USA) in a 50 μl reaction volume as recommended by the manufacturer (Perkin Elmer Cetus, Norwalk, CT, USA). The following amplification conditions were used: 30 cycles of 94°C for 30 s, 62°C for 30 s and 72°C for 1 min, preceded by a 94°C, 10 min primary denaturation step and followed by a 72°C, 7 min final extension step. An aliquot of 1 μl of each product was subsequently diluted 1:20 with the loading buffer (95% formamide, 2 mM EDTA, 0.05% bromophenol blue, 0.05% xylene cyanol) and denatured at 90°C for 5 min. A 2 μl aliquot was loaded onto a 6% acrylamide non-denaturing sequencing gel in 89 mM Tris-borate, 2 mM EDTA pH 8.3 and electrophoresed for 5 h at 4°C at 35 W. Gels were subsequently dried and bands visualised by autoradiography. DNA sequencing was performed on PCR-amplified *nm23-H1* cDNA with the same primers as described above using the DNA cycle sequencing system (BRL, Gaithersburg, MD, USA), according to the manufacturer's instructions.

### Results nm23-H1 *mRNA expression and mutational analysis*

*Nm23-H1* RNA steady-state levels in 48 samples were determined in comparison with β-actin and 18S rRNA by Northern blot analysis (Figure 1). Autoradiographs were semiquantitatively evaluated by densitometry. Representative data shown in Figure 1 indicate that, although both inter- and intra-subtype variations in band intensity for the *nm23-H1* 0.8 kb mRNA levels were observed, there was a trend towards higher expression levels in the high-grade NHL relative to the low-grade NHL. The *nm23-H1* mRNA levels in some of the high-grade NHL were similar to that of the highly NDPK-A-expressing MCF-7 breast adenocarcinoma cells (Figure 1). The variation of *nm23-H1* gene expression in the various lymphoma subtypes may have resulted from mutations in the coding region of the gene. To examine such a possibility, we screened for *nm23-H1* gene mutations in 48 specimens by RT–PCR/SSCP analysis. None of the tumours showed aberrantly migrating bands in SSCP analysis with any of the primer sets used, which spanned the whole coding region. Twenty-four cDNA samples generated by PCR, representing the various lymphoma subtypes studied, were randomly chosen and the coding regions sequenced. None of these specimens harboured a mutation (data not shown).

*Immunohistochemical reactivity of NDPK-A*

Paraffin-embedded, formalin-fixed sections from 106 resected primary lymphomas, well preserved for immunohistochemical analysis, were studied for tumour cell reactivity with the

**Figure 1** Representative Northern blot analysis showing the *nm23-H1* mRNA levels (and their corresponding immunoreactivities) in low-grade and high-grade NHL as well as in Hodgkin's disease. *nm23-H1* expression although heterogeneous, was relatively higher in the high-grade NHL (similar to control MCF-7 expression level) than in the low-grade NHL. Total RNA was hybridised to *nm23-H1*-specific cDNA probe (top) and, as a control, to β-actin (middle). As a loading control, a negative photomicrograph of ethidium bromide-stained 18S rRNA is shown (bottom).

NDPK-A specific antibody 37.6. Both inter- and intra-subtype variations in NDPK-A expression were exhibited in the immunohistochemical stainings (Figure 2), which corroborated the RNA data. Results of the immunohistochemical analyses are summarised in Table I. Staining intensity of neoplastic cells was compared with non-neoplastic surrounding lymphoid tissue. Cells usually showed cytoplasmic staining. Additional nuclear staining was observed in high-grade lymphomas, i.e. LBCL and Burkitt's lymphoma, ALCL and in Hodgkin's cells of mixed cellularity and NS types. In low-grade NHL, staining was restricted to the cytoplasm. Generally, low-grade malignant B-type NHL exhibited only few stained tumour cells; however, mantle cell lymphomas (Figure 2c) exhibited more positive tumour cells than the other low-grade lymphomas (i.e. lymphocytic lymphoma, Figure 2b; lymphoplasmacytoid lymphoma and follicle centre lymphomas). NDPK-A staining intensity was also higher in this group of lymphomas known to have a more aggressive clinical course. Within the rather heterogeneous group of precursor B-lymphoblastic lymphomas, we found low staining intensities in the very immature lymphomas, pre-pre-B and pre-B, whereas more mature lymphoblastic lymphomas presented with considerably more stained tumour cells and a higher staining intensity. Among the other high-grade malignant B-type NHL, almost all tumour cells exhibited strong expression of NDPK-A. The most prominent tumour positivity and staining intensity was observed in Burkitt's lymphoma specimens (Figure 2d and Table I).

In T-cell lymphomas, two tumour sites were investigated: nodal lymphomas and the clinically rather non-aggressive cutaneous lymphomas. In cutaneous T-cell lymphomas (CTCL) fewer immunoreactive cells were observed than in lymph node lymphomas with a similar morphology (e.g. pleomorphic CTCL contained usually lower fractions of

**Figure 2** Immunohistochemical analysis of nm23-H1/NDPK-A protein (**a**–**f**) and Ki-67 antigen (**g**–**i**). (**a**) Benign lymphadenitis. (**b**) Low-grade NHL (B-cell CLL) with relatively weak NDPK-A staining in comparison with another low-grade NHL (mantle cell lymphoma) in (**c**). (**d**) Burkitt's lymphoma tissue (high-grade NHL) with strong NDPK-A staining. (**e**) A T-cell lymphoma tissue (ALCL) exhibiting strong NDPK-A immunoreactivity. (**f**) HD tissue (mixed cellularity) showing very strong NDPK-A positivity of Reed–Sternberg cells. Ki-67 antigen expression is shown in follicle centre lymphoma (follicular) (**g**) precursor B-lymphoblastic lymphoma (**h**) and HD (**i**). Immunohistochemical staining with anti-NDPK-A monoclonal antibody 37.6 and MIB-1 (Ki-67 antigen) monoclonal antibody is described in 'Materials and methods'. Scale bar 50 μm.

reactive cells than the same type of nodal lymphomas). In contrast to high-grade B-cell lymphomas, we did not find striking discrepancies in staining intensity and positivity of cells when looking at T-cell precursor lymphoblastic lymphomas (Table I). Anaplastic large-cell lymphomas (ALCL) behaved like other high-grade lymphomas, usually presenting with high rates of intensely stained tumour cells (Figure 2e). In lymphadenitis, which was used as control, MAb 37.6 reactivity was observed mainly within the cytoplasm of blasts in reactive enlarged germinal centres. (Figure 2a). Centrocytes and mantle cells were usually not stained. Centroblasts and, occasionally, starry-sky macrophages showed the highest reactivity. Within the T zones, single blastic T cells, plasma cells and histiocytes were weakly positive. Small lymphocytes were Mab 37.6 negative.

In Hodgkin's lymphoma, nodular sclerosing (NS) and mixed cellularity (MC) subtypes showed marked positivity of the Hodgkin and Reed–Sternberg cells. These neoplastic cells had the most intense cytoplasmic staining (Figure 2f) when compared with the staining intensities of the different cellular components in high-grade NHL. Cells from the surrounding infiltrates such as lymphocytes or plasma cells showed weak staining (Figure 2f). In the lymphocyte-rich and, strikingly, lymphocyte-depleted subtypes, NDPK-A staining intensity of the neoplastic cells was lower than in both other subtypes of HD.

Children usually present with high-grade lymphomas of the B and T type or Hodgkin's disease. They commonly do not present with low-grade lymphomas. Comparing the results between children (<20 years) and adult cases of each subtype, no differences were observed in either staining patterns or intensity (Table I).

### *Comparison of the proliferative activity and NDPK-A immunostaining in lymphomas*

MIB-1 is a monoclonal antibody that recognises the cell proliferation marker Ki-67 in paraffin-embedded tissues. To determine whether NDPK-A expression correlates with the proliferative activity, we stained all lymphoma specimens with the MIB-1 monoclonal antibody. Figure 2 shows representative Ki-67 antigen immunoreactivities. There was no conistent correlation between NDPK-A activity and proliferative activity (Table I).

### *Correlation between histological subtype and NDPK-A expression*

As exemplified in Figure 2 and summarised in Table I, there was considerable variation in NDPK-A staining intensities among the lymphoma types studied (non-parametric Kruskal–Wallis test; $\chi^2=20.481$, $P=0.0001$). Both high-grade NHL and Hodgkin's lymphoma samples exhibited significantly higher NDPK-A expression levels than low-grade NHL (Wilcoxon's test; $P=0.0002$ and $P=0.0001$ respectively). By contrast, there was no significant difference in NDPK-A expression between high-grade NHL and Hodgkin's lymphoma samples (Wilcoxon's test; $P=0.0529$).

### Discussion

The relationship between *nm23-H1*/NDPK-A and tumour metastatic potential in various human tumours and experimental model systems remains a contentious subject, with conflicting reports from different research groups. Our data demonstrate that *nm23-H1*/NDPK-A is expressed in varying amounts in all lymphomas irrespective of clinical presentation. In contrast to the findings reported for some other tumours such as breast cancer (Bevilacqua *et al.*, 1989; Barnes *et al.*, 1991; Hennessy *et al.*, 1991; Hirayama *et al.*, 1991), in which high *nm23-H1*/NDPK-A expression correlates with non-aggressive disease, our results show that, in lymphomas, high-level *nm23-H1*/NDPK-A expression is found in high-grade subtypes, similar to the situation in Ewing tumours (Aryee *et al.*, 1995), neuroblastomas (Hailat *et al.*, 1991), lung carcinomas (Engel *et al.*, 1993) and prostate cancers (Igawa *et al.*, 1994). These contrasting results might indicate that *nm23-H1* can function as a suppressor gene in some types of cancer and can be associated with tumour aggressiveness in others. This is in agreement with the situation in colorectal carcinomas, in which *nm23-H1* mRNA expression is correlated with increasing colorectal cancer size and extent of local bowel invasion and therefore is speculated to be associated with local aggressive behaviour (Zeng *et al.*, 1994). Contrary to neuroblastomas, in which mutations in the *nm23-H1* gene have also been associated with advanced stages of the disease (Chang *et al.*, 1994), no mutations have so far been found in any of the high-grade lymphomas analysed. Within the group of high-grade B-cell lymphomas, usually strong expression of NDPK-A was observed. In the heterogeneous group of precursor B lymphoblastic lymphomas, the mature types showed relatively higher expression levels than the immature types (pre-pre-B and pre-B). These data might parallel the different clinical behaviour of these tumours as phenotypically mature lymphoblastic B-cell lymphomas require more aggressive and different chemotherapy regimens than the immature lymphomas.

The highest cytoplasmic staining intensity, even compared with the reactivity in high-grade NHL, was observed in HD. NDPK-A antigen was present on Hodgkin and Reed–Sternberg cells, whereas the accompanying plasma cells and lymphocytes showed only faint (if any) staining. In the classical forms of HD (NS, MC and LD), Hodgkin's and Reed–Sternberg cells express activation markers such as CD30, whereas they usually do not express T- or B-cell markers. The high expression levels of NDPK-A by Hodgkin and Reed–Sternberg cells might be interpreted in different ways because of the still enigmatic origin of the Hodgkin and Reed–Sternberg cell. NDPK-A expression paralleled proliferative activity in nodular scleroses and in the mixed cellularity form of HD but not in the lymphocyte-depleted subtype, which is known to have the worst prognosis of all HD subtypes.

The skin is a very common site for extranodal lymphoma origin. Usually, CTCL has a more favourable clinical course and is less aggressive than the T-cell lymphomas arising in lymph nodes. The nodal T-cell lymphomas analysed exhibited slightly higher NDPK-A expression levels compared with primary skin lymphomas of the same tumour morphology. This might reflect a correlation of NDPK-A expression to the clinical course of the disease subtypes. However, as the result of small patient numbers within the individual lymphoma subtypes, statistical evaluation of these small NDPK-A expression differences would be inappropriate and was not done. Although high-grade lymphomas usually have a high proliferative fraction and tend to exhibit more positivity for NDPK-A than low-grade lymphomas, we could not demonstrate a consistent correlation of NDPK-A expression with proliferation, as has been demonstrated for normal lymphoid cells (Keim *et al.*, 1992). There was also considerable intra- and inter-subtype variation of Ki-67 antigen immunoreactivity. Along a gradient among low-grade lymphomas, NDPK-A expression appeared to parallel aggressive clinical course much more than Ki-67 staining (proliferation index), with mantle cell lymphoma exhibiting the highest NDPK-A expression. In addition to a general trend towards *nm23-H1* expression paralleling aggressiveness of the different lymphoma subtypes, intra-subtype variations in *nm23-H1*/NDPK-A were frequently observed in our study. We found neither correlation of NDPK-A expression with patient's age nor sex. As the tumour samples used for this study have been obtained from different centres, and most of the patients involved have been lost for follow-up, no statistical analysis of individual clinical courses in relation to NDPK-A expression was performed. Consequently, though our study underlines the biological importance of *nm23-H1* expression for lymphomas, long term prospective studies with large numbers of patients for each histological subtype are warranted to statistically evaluate the prognostic significance for individual patients.

**Acknowledgements**
This work is dedicated to the memory of Professor Dr T Radaszkiewicz. We appreciate the generosity of Drs Michel Veron, Francois Traincard and Ioan Lascu for the monoclonal antibody 37.6. We also thank Dr Patricia Steeg for the pnm23-H1 and H2 clones. This work was supported in part by the Fonds zur Förderung der Wissenschaftlichen Forschung, grant no. P9238-MED and by the Österreichische Kinderkrebshilfe.

### References

ARYEE DNT, STRÖBEL T, KOS K, SALZER-KUNTSCHIK M, ZOUBEK A, VERON M, AMBROS IM, TRAINCART F, GADNER H AND KOVAR H. (1995). High *nm23-H1*/NDPK-A expression tumours: paradoxical immunohistochemical reactivity and lack of prognostic significance. *Int. J. Cancer*, **64**, 104–111.

BACKER JM, MENDOLA CE, KOVESDI I, FAIRHURST JL, O'HARA B, EDDY JR RL, SHOWS TB, MATHEW S, MURTY VVVS AND CHAGANTI RSK. (1993). Chromosomal localization and nucleoside diphosphate kinase activity of human metastasis suppressor genes *NM23-H1* and *NM23-H2*. *Oncogene*, **8**, 497–502.

BARNES R, MASOOD S, BARKER E, ROSENGARD AM, COGGIN DL, CROWELL T, KING CR, PORTER-JORDAN K, WARGOTZ ES, LIOTTA LA AND STEEG PS. (1991). Low *nm23* protein expression in infiltrating ductal breast carcinomas correlates with reduced patients survival. *Am. J. Pathol.*, **139**, 245–250.

BEVILACQUA G, SOEBEL ME, LIOTTA L AND STEEG PS. (1989). Association of low *nm23* RNA levels in human primary infiltrating ductal breast carcinoma indicators of high tumour metastatic potential. *Cancer Res.*, **49**, 5185–5190.

BIGGS J, HERPERGER E, STEEG PS, LIOTTA L AND SHEARN A. (1990). A *drosophila* gene that is homologous to a mammalian gene associated with tumour metatasis codes for a nucleoside diphosphate kinase. *Cell*, **63**, 933–940.

CHANG CL, XIAO-XIAN Z, THORAVAL DH, UNGAR D, RAWWAS J, HORA N, STRAHLER JR, HANASH SM AND RADANY E. (1994). *Nm23-H1* mutation in neuroblastoma. *Nature*, **370**, 335–336.

CHOMCZYNSKI P AND SACCHI N. (1987). Single-step method of RNA isolation by acid guanidinium thiocyanate–phenol–chloroform extraction. *Anal. Biochem.*, **162**, 156–159.

COHN KH, WANG F, DESOTO-LAPAIX F, SOLOMON WB, PATTERSON LG, ARNOLD MR, WEIMER J, FELDMAN JG, LEVY AT, LEONE A AND STEEG PS. (1991). Association of *nm23-H1* allelic deletions with distant metastases in colorectal carcinoma. *Lancet*, **338**, 722–724.

DAWKINS HJS, GOODALL RJ, HÄHNEL E, SARNA M, ROBBINS PD, DEKLERK N, HÄHNEL R, PAPADIMITRIOU JM, HARVEY JM AND STERRETT GF. (1993). *Nm23-H1* metastasis suppressor gene expression in primary breast cancer. Associations with lymph node status, tumour size, type and grade. *Breast*, **2**, 239–245.

ENGEL M, THEISINGER B, SEIB T, SEITZ G, HUWER H, ZANG KD, WELTER C AND DOOLEY S. (1993). High levels of *nm23-H1* and *nm23-H2* mRNA in human squamous-cell lung carcinoma are associated with poor differentiation and advanced tumour stages. *Int. J. Cancer*, **55**, 375–378.

FLORENES VA, AAMDAL S, MYKLEBOST O, MAELANDSMO M, BRULAND OS AND FOTSTAD O. (1992). Levels of *nm23* RNA in metastatic malignant melanomas. Inverse correlation to disease progression. *Cancer Res.*, **52**, 6088–6091.

GAYNOR ER AND ULTMANN JE. (1984). Non-Hodgkin's lymphoma: management strategies. *N. Engl. J. Med.*, **311**, 1506–1508.

GILLES AM, PRESECAN E, VONICA A AND LASCU I. (1991). Nucleoside diphosphate kinase from human erythrocytes. *J. Biol. Chem.*, **266**, 8784–8789.

HAILAT N, KEIM DR, MELHEM RF, ZHU XX, ECKERSKORN C, BRODEUR GM, REYNOLDS CP, SEEGER RC, LOTTSPEICH F, STRAHLER JR AND HANASH SM. (1991). High levels of p19/*nm23* protein in neuroblastoma are associated with advanced stage disease and with *n-myc* gene amplification. *J. Clin. Invest.*, **88**, 341–345.

HARRIS NL, JAFFE ES, STEIN H, BANKS PM, CHAN JKC, CLEARY ML, DELSOL G, WOLF-PEETERS CD, FALINI B, GATTER KC, GROGAN TM, ISAACSON PG, KNOWLES DM, MASON DY, MULLER-HERMELINK H-K, PILERI SA, PIRIS MA, RALFKIAER E AND WARNKE RA. (1994). A revised European-American classification of lymphoid neoplasms: a proposal from the International Lymphoma Study Group. *Blood*, **84**, 1361–1392.

HENNESSY C, HENRY JA, MAY FEB, WESTLEY BR, ANGUS B AND LENNARD TWJ. (1991). Expression of the antimetastatic gene *nm23* in human breast cancer: an association with good prognosis. *J. Natl Cancer Inst.*, **83**, 281–285.

HIRAYAMA R, SAWAI S, TAGAKI Y, MISHIMA Y, KIMURA N, SHIMADA N, ESAKI Y, KURASHIMA C, UTSUYAMA M AND HIROKAWA K. (1991). Positive relationship between expression of anti-metastatic factor (*nm23* gene product of nucleoside diphosphate kinase) and good prognosis in human breast cancer. *J. Natl Cancer Inst.*, **83**, 1249–1250.

HORNING SJ AND ROSENBERG SA. (1984). The natural history of initially untreated low-grade non-Hodgkin's lymphomas. *N. Engl. J. Med.*, **98**, 1471–1475.

IGAWA M, RUKSTALIS DB, TANABE T AND CHODAK GW. (1994). High levels of *nm23* expression are related to cell proliferation in human prostate cancer. *Cancer Res.*, **54**, 1313–1318.

KATH R, DONHUIJSEN K, HAYUNGS J, ALBRECHT K, SEEBER S AND HÖFFKEN K. (1995). Primary gastric non-Hodgkin's lymphoma: a clinicopathological study of 41 patients. *J. Cancer Res. Clin. Oncol.*, **121**, 51–56.

KEIM D, HAILAT N, MELHEM R, ZHU XX, LASCU I, VERON M AND STRAHLER JJ. (1992). Proliferation-related expression of p19/*nm23* nucleoside diphosphate kinase. *J. Clin. Invest.*, **89**, 919–924.

LACOMBE ML, WALLET V, TROLL H AND VERON M. (1990). Functional cloning of a nucleoside diphosphate kinase from *Dictyostelium discoideum*. *J. Biol. Chem.*, **265**, 10012–10018.

LEONE A, FLATOW U, RICHTER-KING C, SANDEEN MA, MARGULIES IMK, LIOTTA L AND STEEG PS. (1991). Reduced tumour incidence, metastatic potential, and cytokine responsiveness of *nm23*-transfected melanoma cells. *Cell*, **65**, 25–35.

LEONE A, FLATOW U, VANHOUTTE K AND STEEG PS. (1993*a*). Transfection of human *nm23-H1* into the human MDA-MB-435 breast carcinoma cell line: effects on tumour metastatic potential, colonization and enzymatic activity. *Oncogene*, **8**, 2325–2333.

LEONE A, SEEGER RC, HONG CM, HU YY, ARBOLEDA MJ, BRODEUR GM, STRAM D, SLAMON DJ AND STEEG PS. (1993*b*). Evidence for *nm23* RNA overexpression, DNA amplification and mutation in aggressive childhood neuroblastomas. *Oncogene*, **8**, 855–865.

NAKAYAMA T, OHTSURU A, NAKAO K, SHIMA M, NAKATA K, WATANABE K, ISHII N, KIMURA N AND NAGATAKI S. (1992). Expression in human hepatocellular carcinoma of nucleoside diphosphate kinase, a homologue of the *nm23* gene product. *J. Natl Cancer Inst.*, **84**, 1349–1354.

POSTEL EH, BERBERICH SJ, FLINT SJ AND FERRONE CA. (1993). Human c-myc transcription factor Puf identified as *nm23-H2* nucleoside diphosphate kinase, a candidate suppressor of tumour metastasis. *Science*, **261**, 478–480.

ROSENGARD AM, KRUTZSCH HC, SHEARN A, BIGGS JR, BARKER E, MARGULIES IMK, KING CR, LIOTTA LA AND STEEG PS. (1989). Reduced *Nm23*/Awd protein in tumour metastasis and aberrant Drosophila development. *Nature*, **342**, 177–180.

SASTRE-GARAU X, LACOMBE ML, JOUVE M, VERON M AND MAGDELENAT H. (1992). Nucleoside diphosphate kinase/NM23 expression in breast cancer: lack of correlation with lymph-node metastasis. *Int. J. Cancer*, **50**, 533–538.

STAHL JA, LEONE A, ROSENGARD AM, PORTER L, KING CR AND STEEG PS. (1991). Identification of a second human nm23 gene, nm23-H2. *Cancer Res.*, **51**, 445–449.

STEEG PS, BEVILACQUA G, KOPPER L, THORGEIRSSON UP, TALMADGE JE, LIOTTA LA AND SOEBEL ME. (1988). Evidence for a novel gene associated with low tumour metastatic potential. *J. Natl Cancer Inst.*, **80**, 200–204.

ZENG ZS, HSU S, ZHANG ZF, COHEN AM, ENKER WE, TURNBULL AA AND GUILLEM JG. (1994). High level of Nm23-H1 gene expression is associated with local colorectal cancer progression not with metastases. *Br. J. Cancer*, **70**, 1025–1030.